# A rare pathology that mimics lung cancer: IgG4‐related vasculitis

**DOI:** 10.1002/rcr2.70039

**Published:** 2024-10-10

**Authors:** Coskun Ardan Sener, Aylin Ozgen Alpaydın, Oguz Kılınc

**Affiliations:** ^1^ Department of Pulmonary Diseases Dokuz Eylül University Faculty of Medicine Hospital Izmir Turkey; ^2^ Department of Pulmonary Diseases Economy University Faculty of Medicine Hospital Izmir Turkey

**Keywords:** biopsy, IgG4, lung, mass, vasculitis

## Abstract

Immunoglobulin‐G4 (IgG4)‐related disease is essentially a fibro‐inflammatory disease that can affect any organ simultaneously or at different times. The disease usually presents with organ growth that mimics a tumour and can affect the lacrimal glands, major salivary glands, pancreas, bile ducts, retroperitoneal area, lungs, kidneys, aorta, meninges and thyroid gland. The immunopathogenesis behind this new disease has not yet been elucidated. Histopathological distinguishing features of the disease include dense lymphoplasmocytic infiltrates dominated by IgG4 positive plasma cells, storiform fibrosis and obliterative phlebitis. The likelihood of developing with immunoglobulin G4 (IgG4‐RD) is a recently identified rare systemic fibroinflammatory disease with an estimated incidence of less than 1 in 100,000 people worldwide. We present our case, which was diagnosed with IGG4‐related vasculitis by lung fine needle aspiration biopsy, which is very rare in the literature.

## Graphical abstract

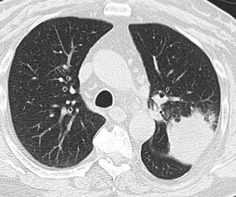

## INTRODUCTION

Immunoglobulin G4 (IgG4) is the lowest among the IgG subfractions. IgG4 is believed to be different from other antibodies and its level in serum is between 0.35 and 0.51 mg/mL.^1^ IgG4 does not have an activating effect on complement pathways and binds to Fcγ receptors at a lower percentage than other IgG subclasses.^1^, ^2^ The biggest problem in diagnosis is IgG4‐related disease can often present with findings that mimic malignancy both clinically and radiologically. For this reason it is very important to accurately and timely differentiate IgG4‐related disease from a malignant tumour of the affected organ (cancer or lymphoma) to avoid erroneous diagnosis of malignancy.^2^ Inflammatory conditions, including many chronic rheumatic diseases, may also be linked to increased IgG4 levels.^1^, ^2^, ^3^, ^4^ It is also characterized by storiform fibrosis in pathological diagnosis and obliterative phlebitis, and can clinically affect almost every organ of the body.^3^ Common clinical manifestations of the disease involves enlargement of the lacrimal and major salivary glands, various forms of lung diseases, autoimmune pancreatitis, sclerosing cholangitis, tubulointerstitial nephritis and retroperitoneal fibrosis.^4^ Clinical symptoms of lung involvement of the disease are non‐specific and may manifest itself with complaints such as cough, shortness of breath, chest pain and fever. Inflammatory pseudotumors of the lung may occur, which contain IgG4 plasma cells with disease fibrosis and are characterized by obliterative phlebitis.^4^

## CASE REPORT

**FIGURE 1:**
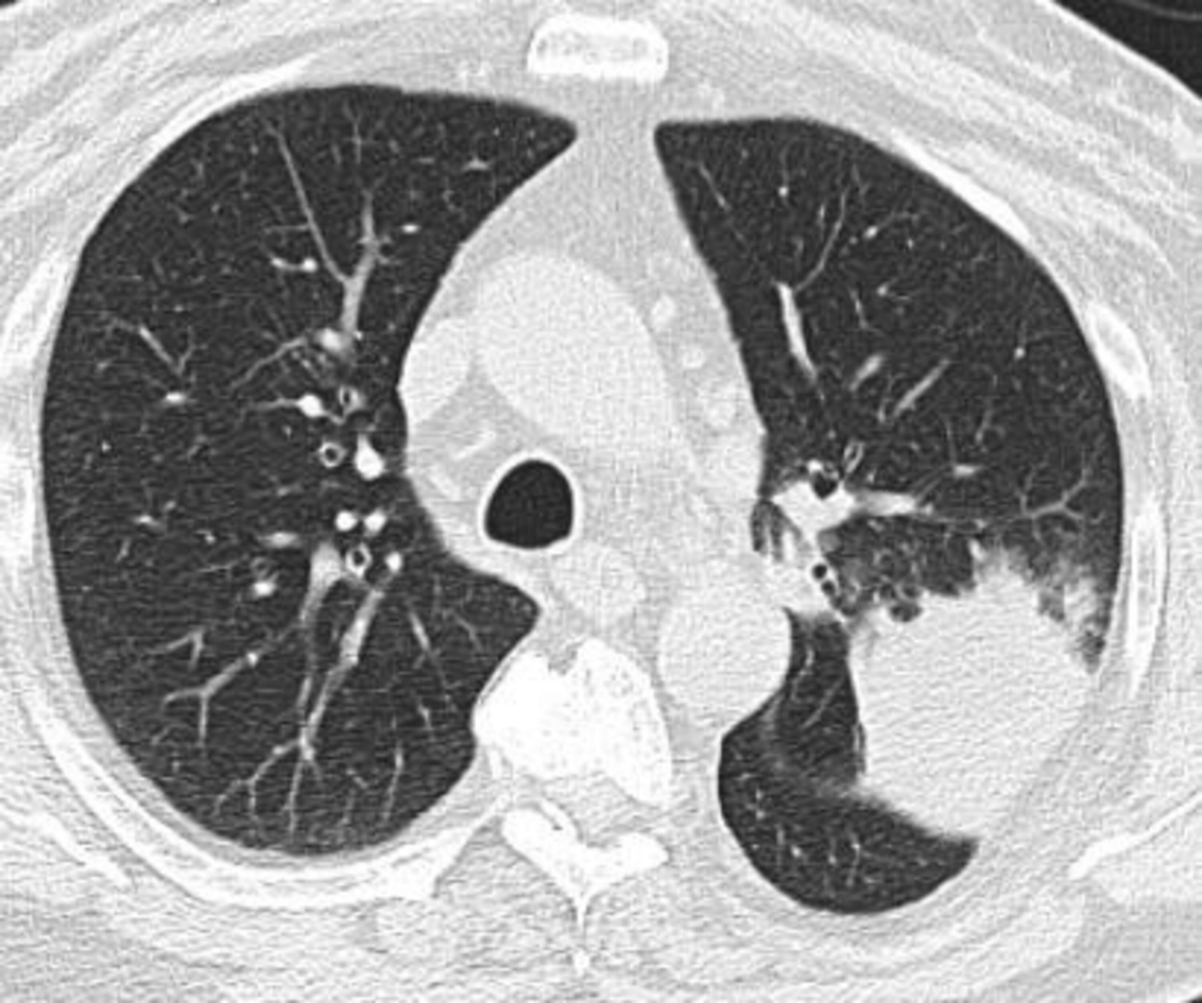
Mass lesion in the upper lobe of the left lung.

**FIGURE 2:**
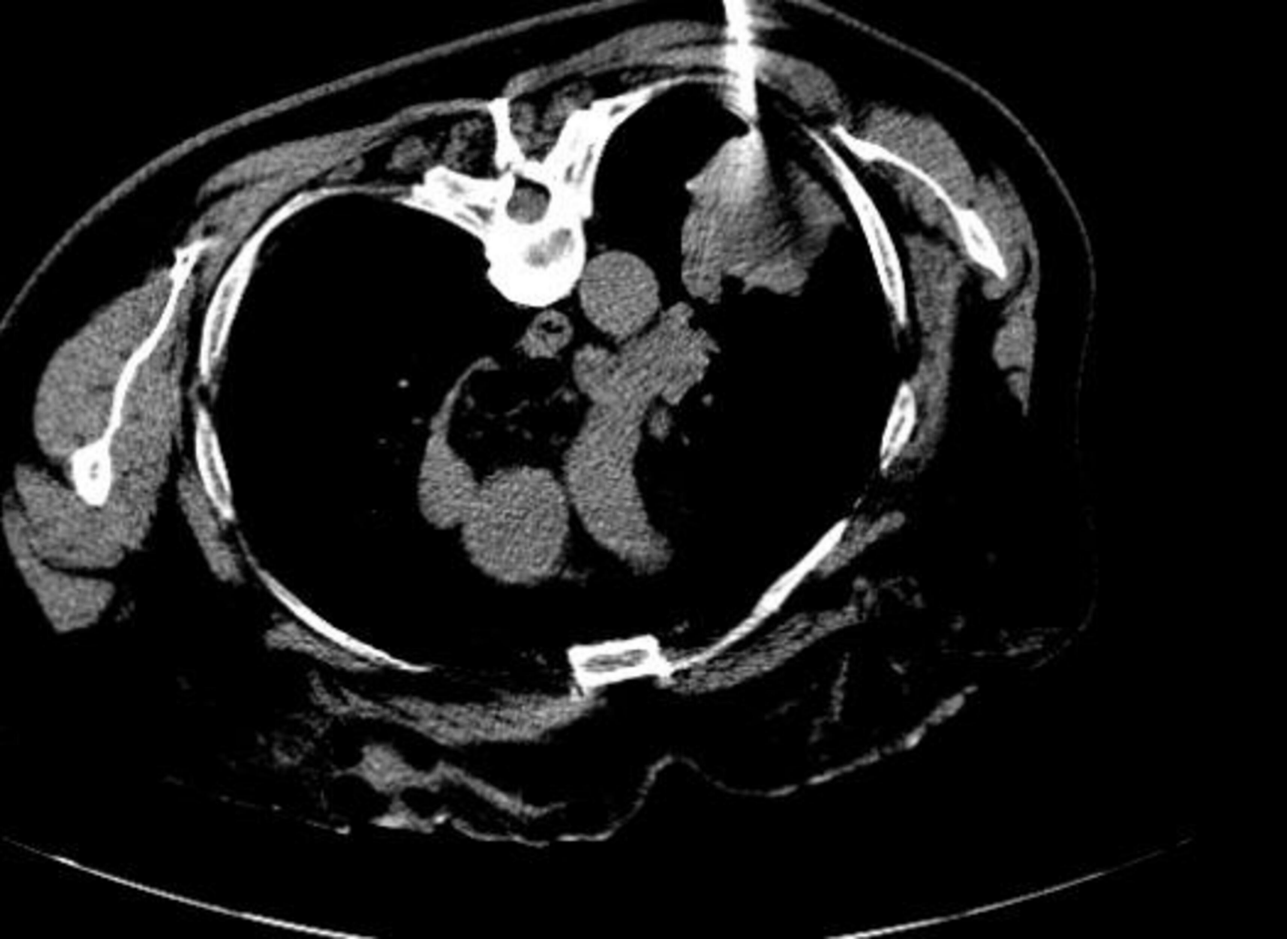
Core needle biopsy from the mass lesion in the upper lobe of the left lung.

A 66‐year‐old male patient presented to the chest diseases outpatient clinic with complaints of hemoptysis of up to two water glasses at a time and an intermittent dry cough, which occurred 20 days before his admission. There were no other active pulmonary complaints. His vital signs were stable on admission. From his history, it was learned that he quit smoking 5 years previously and that he had smoked 20 cigarettes a day before then. In the thorax computed tomography taken 20 days before his application, an image compatible with a 4.5 × 4.2 cm mass was detected in the upper lobe of the left lung (Figure 1). The patient underwent core needle biopsy (Figure 2). The biopsy result of the patient was evaluated as IgG4 expression in most of the IgG positive cells. As far as can be evaluated, the IgG4/IgG ratio was reported to be over 40%. After the patient's diagnosis was finalized, his treatment was planned together with the rheumatology department. Azotiopirin 200 mg/day and methylprednisolone 90 mg/day were planned as initial treatment. In maintenance treatment, it was planned to reduce the methylprednisolone dose by 8 mg every 10 days. The patient has been receiving treatment for one and a half month. The patient's hemoptysis stopped after the 3rd week after treatment and his cough complaint disappeared. The patient will undergo a control thorax CT 3 months after treatment.

## DISCUSSION

Elevated IgG4 may be found in malignancies, allergic disorders, and parasitic infections. In the inflammatory phase of the disease, with the activation of the above‐mentioned antigens, granzyme and perforin are secreted, especially from CD4^+^ cytotoxic T lymphocytes. In addition, cytokines that play a role in fibrosis, such as IL‐1B, IL‐6, TGF‐beta and interferon gamma are released. The disease generally responds well to steroids, but occasionaly relapse can occur, especially in patients with high risk factors. IgG4‐IH is accepted as a multisystemic disease with a wide spectrum that can also involve the eye, biliary tract, aorta, parotid and submandibular glands, pancreas, kidney, lung and pituitary. While a history of allergic disease, Mikulicz syndrome (sialadenitis and dacryoadenitis) and thyroiditis are more common in women. Autoimmune pancreatitis, retroperitoneal fibrosis and sclerosing cholangitis are more common in men. While the clinical findings of the disease can be seen as masses, hypertrophy and organomegaly in the affected organs, it can also occur with radiological and histological findings detected incidentally without clinical symptoms.^1^ Studies have shown that the disease can also involve vessels such as the aorta, coronary artery and subclavian artery.^2^, ^3^ The presence of ANCA positivity can be observed in IgG4‐IH patients and, conversely, the presence of high IgG4 serum levels in ANCA‐related vasculitis. Mild eosinophilia is common in patients with both IgG4‐IH and granulomatous polyangiitis.^2^ Although lung involvement in IgG4‐related disease is more common in middle‐aged or elderly men, the average age in men was found to be 69 years old. The disease includes the interstitium and mediastinum in the lung. It can involve the airways and the pleura. The disease can also cause acute interstitial pneumonia involvement on chest computed tomography, characterized by honeycomb formation bilaterally, predominantly in the lower lobes. Involvement compatible with organizing pneumonia can also be observed in the disease. At the same time, unilateral pleural effusion can rarely be observed. Bilateral non‐hilar adenopathy is also a common finding. In thorax computed tomography, bronchovascular thickening bundles and prominent interlobular septae, mass or large nodular lesions can be seen. Diagnosis of the disease is based on two criteria: serum IgG4 concentration greater than 135 mg/dL; or IgG4+/IgG plasma cell ratios should be over 40%. In the treatment of this disease, a dose of 0.6 mg/kg/day steroid is given for 2–4 weeks, decreasing by 5 mg/day every 2 weeks. However, higher doses of steroids are required to maintain remission in lung involvement of the disease.^4^ Studies have found that more successful results were obtained in cases receiving steroid treatment. Immunosuppressant agents such as azathioprine, mycophenolate mofetil and cyclosporine can be used in treatment.^3^, ^4^

## AUTHOR CONTRIBUTIONS

Coşkun Ardan Şener, Aylin Özgen Alpaydın, and Oǧuz Kılınç were involved in patient care. All authors were involved in preparation of the manuscript and images. All authors approved the final manuscript.

## CONFLICT OF INTEREST STATEMENT

None declared.

## ETHICS STATEMENT

The authors declare that appropriate written informed consent was obtained for the publication of this manuscript and accompanying images.

## Data Availability

The data that support the findings of this study are available on request from the corresponding author. The data are not publicly available due to privacy or ethical restrictions.
